# Zinc Stabilizes Shank3 at the Postsynaptic Density of Hippocampal Synapses

**DOI:** 10.1371/journal.pone.0153979

**Published:** 2016-05-04

**Authors:** Jung-Hwa Tao-Cheng, Dana Toy, Christine A. Winters, Thomas S. Reese, Ayse Dosemeci

**Affiliations:** 1 NINDS EM Facility, National Institute of Neurological Disorders and Stroke, National Institutes of Health, Bethesda, MD, United States of America; 2 Laboratory of Neurobiology, National Institute of Neurological Disorders and Stroke, National Institutes of Health, Bethesda, MD, United States of America; University of Sydney, AUSTRALIA

## Abstract

Shank3 is a postsynaptic density (PSD) scaffold protein of the Shank family. Here we use pre-embedding immunogold electron microscopy to investigate factors influencing the distribution of Shank3 at the PSD. In dissociated rat hippocampal cultures under basal conditions, label for Shank3 was concentrated in a broad layer of the PSD, ~20–80 nm from the postsynaptic membrane. Upon depolarization with high K^+^ (90 mM, 2 min), or application of NMDA (50 μM, 2 min), both the labeling intensity at the PSD and the median distance of label from the postsynaptic membrane increased significantly, indicating that Shank3 molecules are preferentially recruited to the distal layer of the PSD. Incubation in medium supplemented with zinc (50 μM ZnCl_2_, 1 hr) also significantly increased labeling intensity for Shank3 at the PSD, but this addition of Shank3 was not preferential to the distal layer. When cells were incubated with zinc and then treated with NMDA, labeling intensity of Shank3 became higher than with either treatment alone and manifested a preference for the distal layer of the PSD. Without zinc supplementation, NMDA-induced accumulation of Shank3 at the PSD was transient, reversing within 30 min after return to control medium. However, when zinc was included in culture media throughout the experiment, the NMDA-induced accumulation of Shank3 was largely retained, including Shank3 molecules recruited to the distal layer of the PSD. These results demonstrate that activity induces accumulation of Shank3 at the PSD and that zinc stabilizes PSD-associated Shank3, possibly through strengthening of Shank-Shank association.

## Introduction

Shank family proteins are coded by three genes (Shank1, 2 and 3) and represent an important category of scaffold proteins at the postsynaptic density (PSD) [1]. Shanks are involved in aspects of synaptic development and function [2], and Shank3 was the first to be implicated in autism spectrum disorders [3]. Studies with dissociated cell cultures indicated a role for Shank3 in the formation of functional dendritic spine synapses [4], and other studies using mouse models linked mutations of Shank3 to synaptic dysfunction and behavioral abnormalities [5, 6].

Redistribution of Shanks at the PSD under excitatory conditions was documented by immunogold electron microscopy (EM), using a pan-Shank antibody that recognizes all three Shanks as well as antibodies specific for Shank1 or Shank2 [7–9]. However, specific information about redistribution of Shank3 is only now attainable with the recent availability of suitable antibodies for immunogold EM. The present study focuses on the redistribution of Shank3 at the PSD under different experimental conditions, with a special emphasis on the effect of zinc.

Zinc is prevalent at synapses, and its deficiency or excess has been associated with pathological conditions, including neurological diseases [10–13]. Zinc is also present in PSD fractions and is important for the structural integrity of the isolated PSD [14]. It is of particular interest that zinc binds tightly to the SAM domain of Shank3, and regulates its assembly *in vitro* [15]. Studies by light microscopy show concerted action of zinc and Shank3 in synaptogenesis and synapse maturation [16], and increased labeling intensity of Shank3 at synaptic puncta upon incubation with zinc [17].

Here, we use pre-embedding immunogold EM to document the labeling intensity as well as the laminar distribution of Shank3 within the PSD to characterize the effects of excitatory stimuli and zinc supplementation. Shank3 distribution after cessation of NMDA treatment was further investigated to identify the role of zinc in retaining Shank3 at the PSD.

## Materials and Methods

### Antibodies

Shank3 ab1 [Rabbit polyclonal antibody against Shank3 (aa 1431–1590 of human Shank3; 1:200 for Western; 1:50 for EM)] was from Santa Cruz (Dallas, Texas). Specificity of this antibody is shown in [18], where knocking down Shank3 in hippocampal cultures via RNA interference significantly reduces the level of Shank3 but not Shank1 or Shank2. Shank3 ab1 yielded clear and specific labeling at PSDs in perfusion-fixed mouse brains, but gave an unexpectedly noisy signal in dissociated rat hippocampal cell cultures. In addition to PSDs, this antibody also heavily labeled lysosomes and extracellular matrix in cell cultures (images not shown). However, within the synaptic region, labeling was specifically localized to the PSD.

Shank3 ab2 [Rabbit polyclonal antibody against Shank3 (aa 1055–1616 of rat Shank3; 1:1000 for Western; 1:200–800 for EM)] was from Synaptic Systems (Goettingen, Germany). Specificity of this antibody was tested by immunoblotting, with blocking by pre-adsorption of the immunogen. When used on cells transfected with the corresponding protein fragments from Shank1 and 2, this Shank3 antibody showed no signal (personal communication with Henrik Martens at Synaptic Systems). Shank3 ab2 labeling was clean in dissociated neuronal cultures, and yielded a virtually identical distribution pattern at the PSD as ab1, except with a much higher labeling intensity than with ab1. Both Shank3 ab1 and ab2 were raised against peptides corresponding to regions of Shank3 with relatively low similarity to Shank1 and Shank2.

Shank3 ab3 [mouse monoclonal against Shank3 (aa 538–626 of rat Shank3; 1:50 for EM), clone N367/62] and Shank3 ab4 [mouse monoclonal against Shank3 (aa 840–857 of rat Shank3; 1:50 for EM), clone N69/46] were from NeuroMab (Davis, CA). Specificity of these antibodies was validated by immunoblotting of COS cells transfected with Shank1, 2, and 3. Furthermore, ab3 was validated with wild type and Shank3 knock-out animals. However, labeling efficiency for ab3 and ab4 was very low in dissociated rat hippocampal cultures where only about 10–15% of the PSDs were credibly labeled, precluding accurate measurement. However, both antibodies yielded credible and specific labeling of PSDs in mouse brain.

Mouse monoclonal antibody against Shank1 (clone N22/21, 1:50–100 for EM), Shank2 (clone N23B/6, 1:1000 for Western), and pan-Shank (clone N23B/49, which recognizes all three members of the Shank family: Shank1, 2 and 3, used at 1:250 for EM) were from NeuroMab (Davis, CA). Mouse monoclonal antibody against alpha-CaMKII (clone 6G9(2), 1:100 for EM) was from Millipore (Billerica, MA).

### Subcellular fractionation, electrophoresis and immunoblotting

Brains from Sprague-Dawley rats were custom collected by Pel-Freeze Biologicals (Rogers, AR) or Rockland (Gilbertsville, PA) within two minutes of decapitation and immediately frozen in liquid nitrogen. Subcellular fractionation to obtain synaptosome and PSD fractions was performed as in [19]. SDS-PAGE was carried out on 4–15% gradient Mini PROTEAN TGX precast polyacrylamide gels (Bio-Rad). After transfer, PVDF membranes were blocked, incubated with specified primary antibodies and then with secondary antibodies (1:50,000 dilution). Immunoblots were visualized by chemiluminescence (SuperSignal West Pico, Thermo Scientific, Waltham, MA).

### Perfusion fixation of mouse brain

The animal protocol was approved by the National Institute of Neurological Disorders and Stroke/National Institute of Deafness and Communication Disorders/National Center for Complementary and Integrative Health Animal Use and Care Committee (Protocol Number: ASP1206) and conforms to NIH guidelines. Three C57BL/6 adult male mice from Biological Testing Branch/National Cancer Institute (Frederick, MD, USA) and one C57BL/6 adult male mouse from Taconic Farms were perfusion fixed. Perfusion fixation was performed as described in [20]. Adult mice were deeply anesthetized with isoflurane, and intracardially perfusion fixed with 4% paraformaldehyde (EMS, Fort Washington, PA) in PBS. Perfusion pressure was maintained at 150 mm Hg with a Perfusion-One System (MyNeurolab, Maryland Heights, MO). Approximately 100 ml of fixative was used per mouse over the course of 10 min. The fixed brain was dissected and immersed in fixative until ready for further slicing. In order to avoid over-fixation, total fixation time was 40–60 min based on the beginning of flow of the fixative into the heart until the time the brain was vibratomed into 100 μm thick coronal slices. The brain slices were stored in PBS at 4˚C for no more than a week before they were immuno-labeled, free-floating in 24-well cell culture plates. Selected areas of the brains, including cerebral cortex, hippocampus and cerebellum, were sampled.

### Dissociated rat hippocampal neuronal cultures and treatment

All animals were housed in an NIH intramural research program vivarium in individually ventilated cages with a 12:12 light:dark cycle. Animals were provided autoclaved hardwood chip bedding which was changed weekly. They were given an autoclaved Prolab RMH 1800 diet and reverse-osmosis water ad lib. Seventeen Sprague Dawley timed pregnant rats from Taconic Farms (Germantown, MD, USA) and Charles River (Raleigh, NC, USA) were used. Pregnant dams were euthanized by CO2 inhalation. Embryos were then collected by caesarian section and decapitated with sharp scissors.

Cell cultures were prepared from embryonic 20 day-old rat fetuses (Animal protocol Number: ASP1159) by papain dissociation, and then plated onto previously prepared rat glial feeder cultures. Cultures were maintained in MEM Eagle Salts containing 3.5 gm/L sodium bicarbonate and 6 gm/L glucose (Invitrogen A14518-01). The media was supplemented with 2 mM Glutamax 1, stabilized form of L-glutamine, L-alanyl-L-glutamine, 5% horse serum and 2% fetal bovine serum, as well as a growth factor cocktail N3 containing apotransferrin, insulin, selenium, T3, putrescine, progesterone, and corticosterone. The cultures were kept in 10% CO_2_ incubator at 35˚C, and experiments were carried out with three week-old cultures.

For high K^+^ experiments, culture dishes were placed on a floating platform in a water bath maintained at 37˚C. Control incubation medium was: 124 mM NaCl, 2 mM KCl, 1.24 mM KH_2_PO_4_, 1.3 mM MgCl_2_, 2.5 mM CaCl_2_, 30 mM glucose in 25 mM HEPES at pH 7.4. High K^+^ medium was at 90 mM KCl, with osmolarity compensated by reducing the concentration of NaCl. Cell cultures were washed with control medium and treated for 2 min with either control or high K^+^ media, and then fixed immediately.

Zinc experiments commenced with changing the media to the serum-free media MEM Eagle Salts supplemented only with 1 mM Glutamax 1, and 1 mM sodium pyruvate (Invitrogen) with or without 50 μM zinc (Sigma 39059 0.1M ZnCl_2_ solution) for 1 hour in the incubator. The cultures were then treated with 50 μM NMDA for 2 min with or without zinc. In some experiments, 50 μM APV, an NMDA blocker (Tocris, Bristol, United Kingdom), was included throughout. After NMDA treatment, cultures were either fixed immediately or incubated further in media without NMDA. Two NMDA washout protocols were carried out: (1) samples were washed five times with the culture media (with or without zinc) and then left in the incubator for a total of 30 min, or (2) samples were washed five times with HEPES-based, calcium-free Ringer containing 1 mM EGTA for a total of 5 min. Cells were fixed with 4% paraformaldehyde in PBS for 25–35 min (25 min for ab1, and 30–35 min for ab2) at room temperature, thoroughly washed with PBS, and stored at 4˚C for no more than 1 wk.

Zinc concentration at 50 μM was determined to be optimal in producing consistent and measurable differences in Shank3 labeling at the PSD after assessing dosages at 10, 25, 50 and 100 μM. The zinc-supplemented cells looked healthy after 1 hr of incubation, and no structural differences were detected from control samples, even at 100 μM zinc. On the other hand, we were not able to successfully use a zinc-chelator, TPEN, to further deplete the zinc concentration because a typical treatment protocol (20 min incubation at 10 μM) resulted in subtle signs of structural damage in neuronal processes, especially in cells older than 3 wks in culture.

### Pre-embedding immunogold labeling and electron microscopy

All steps were carried out at room temperature unless otherwise indicated. Samples were made permeable and blocked with 0.1% saponin and 5% normal goat serum in PBS for 40–60 min, incubated with primary and then secondary antibodies (Nanogold, at 1:200, Nanoprobes, Yaphand, NY) for 1 hr, fixed with 2% glutaraldehyde in PBS for 30 min, and stored at 4˚C in fixative for up to 2 wks. Samples were washed thoroughly in deionized water, silver enhanced (HQ kit, Nanoprobes), treated with 0.2% osmium tetroxide in 0.1M phosphate buffer at pH 7.4 for 30 min, en block stained with 0.25% uranyl acetate in acetate buffer at pH 5.0 for 1 hr at 4˚C, dehydrated in graded ethanols, and embedded in epoxy resin.

### Morphometry

**Fig 1:**
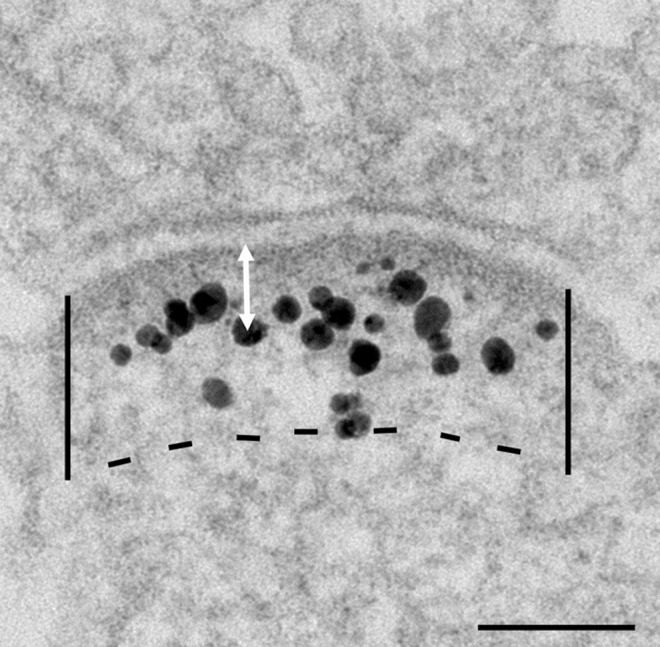
Electron micrograph of an asymmetric synapse from dissociated hippocampal culture labeled for Shank3 with ab2. Labeling intensity at the PSD is measured by counting all particles within the marked area 120 nm below the postsynaptic membrane, divided by the length of the PSD, and expressed as number of labels/μm PSD. Distance of label from the postsynaptic membrane is measured from the center of the particle to the outer edge of the membrane (white arrow). Scale bar = 0.1 μm.

Asymmetric synapses manifested the structural characteristics of presynaptic terminals with clustered synaptic vesicles, rigid appositions of the pre- and post-synaptic membranes forming a synaptic cleft, and a prominent postsynaptic density (PSD) at the postsynaptic membrane [21] (Fig 1). Every cross-sectioned asymmetric synapse encountered in randomly selected grid openings was photographed for morphometry. The PSD is a complex consisting of a ~30 nm layer of dense material immediately beneath the postsynaptic membrane and a distal layer that can extend as deep as ~120 nm into the cytoplasm. The distal layer is less dense and not easily detectible without immunogold labeling for specific PSD scaffold proteins such as Shank [7] or Homer [22]. Here we will use the term PSD to designate the PSD complex.

To assess the amount of Shank3 label at PSDs, the intensity of labeling in PSD extending 120 nm from the postsynaptic membrane was measured (Fig 1) [7, 9] and expressed as number of labels per running μm of PSD length. Labeling intensities among different conditions within each experiment were compared by one-way ANOVA with Tukey’s post-test. Due to variability in labeling efficiency among different experiments, values were normalized to controls for each experiment, and then averaged among experiments. All EM panels in each figure were selected from the same experiment.

To assess the laminar distribution of Shank3 at PSDs with respect to the postsynaptic membrane, the distance from the center of the silver-enhanced gold particle to the outer edge of the postsynaptic membrane was measured for every particle in the marked PSD area (Fig 1), and data from all PSDs was compiled into histograms for each experiment. Because the laminar distribution of Shank3 was typically skewed, values for median instead of mean were used for a nonparametric statistical test (Wilcoxon test; KaleidaGraph, Synergy Software, Reading, PA).

The curvature of a PSD is expressed as an index [20]. The outline of the PSD was traced and a line was drawn between the two ends of the PSD as the chord. The height of this arc was measured and divided by the length of the chord, and converted to a percentage. Micrographs for measurement were oriented so that presynaptic terminals were consistently situated at the top of the PSD. A curvature of zero indicates a flat PSD, which does not arch up or down. PSDs that arch up into the presynaptic terminal are given positive values, whereas those with negative values arch down into the postsynaptic dendrite.

## Results

### Characterization of Shank3 antibodies

**Fig 2:**
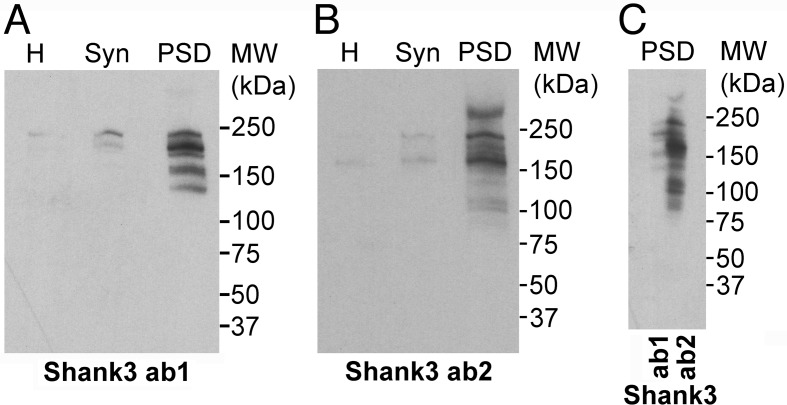
**(A, B)** Western immunoblots with two antibodies for Shank3 (ab1 and ab2) comparing homogenate (H), synaptosome (Syn), and PSD fractions from cerebral cortex of rat show significant enrichment of Shank3 in the PSD fraction. Ten micrograms of protein was loaded into each lane for the homogenate and synaptosome, and five micrograms of protein was loaded for the PSD fraction. **(C)** Western immunoblots with the two Shank3 antibodies reveal a common set of bands in the PSD fraction. After transfer, the membrane was cut in the middle of the lane to separately probe with the two antibodies.

Two antibodies for Shank3 (ab1 and ab2) labeled a common group of electrophoretic bands in the PSD fraction within an apparent molecular weight range of ~140–240 kDa. Both antibodies indicated distinct enrichment of all Shank3 isoforms in the PSD fraction (Fig 2A and 2B). Notably, there are additional bands recognized by Shank3 ab2 (Fig 2C), most likely due to the longer peptide sequence used as immunogen for producing this antibody.

### Distribution of Shank3 in perfusion-fixed mouse brain

**Fig 3:**
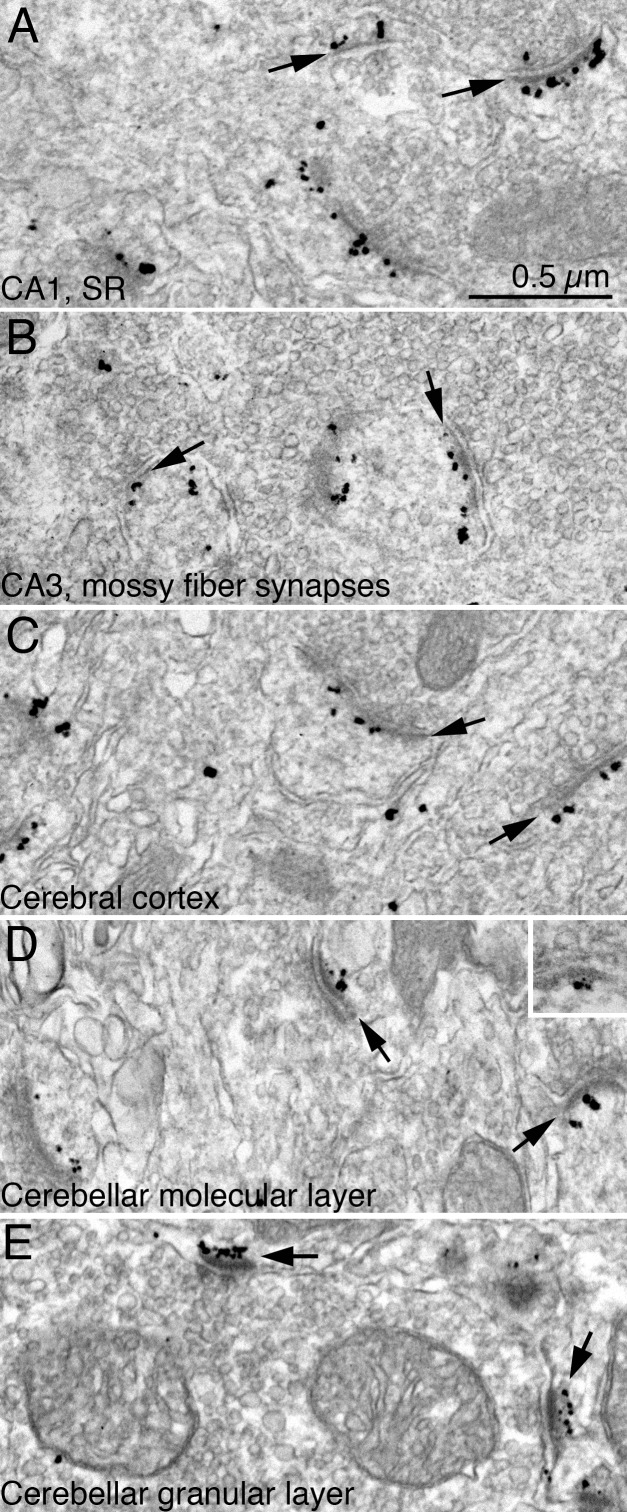
Electron micrographs from perfusion fixed-mouse brain showing immunogold labeling for Shank3 using ab1 (A-E) and ab3 (D, inset). PSDs (arrows) were labeled in all regions examined, including hippocampus [stratum radiatum (SR) of the CA1 region (A), mossy fiber synapses in the CA3 region (B)], cerebral cortex (C), and the outer molecular layer (D) and granular layer (E) of the cerebellum. Scale bar = 0.5 μm.

Consistent with a post-embedding immunogold EM study on rat hippocampus [23], the present pre-embedding immunogold EM shows that Shank3 is specifically localized at PSDs in the CA1 and CA3 regions of mouse hippocampus (Fig 3A and 3B), as well as in the cerebral cortex (Fig 3C) and cerebellum–both at Purkinje spines in molecular layer (Fig 3D) and at PSDs of granule cells in granular layer (Fig 3E).

It is surprising that the PSDs of cerebellar Purkinje spines label for Shank3 because in situ hybridization has shown that Shank3 mRNA is present in the granule cells but not in Purkinje cells [24, 25, 26]. Light microscopy studies show immunolabeling of Shank3 and its binding partner, ProSAPiP1, in the molecular as well as the granular layer of the rat cerebellum [27, 28]. The present EM data using four Shank3 antibodies [ab1 (Fig 3D), ab2 (image not shown), ab3 (Fig 3D inset), and ab4 (image not shown)], which show Shank3-labeled PSDs in both layers of the cerebellum, are consistent with these LM studies. Also, it is the Purkinje spines but not the presynaptic terminals from granule cells that label for Shank3 in the molecular layer of the cerebellum. Furthermore, cerebellar tissue labeled with a pan-Shank antibody that recognizes Shank1, 2 and 3 also specifically labeled PSDs at Purkinje spines but not the axons of granule cells (images not shown). The discrepancy between localization of Shank3 mRNA and Shank3 protein labeling could be due to differential levels of detection by different techniques.

### Activity-induced recruitment of Shank3 is preferential to the distal layer of the PSD

**Fig 4:**
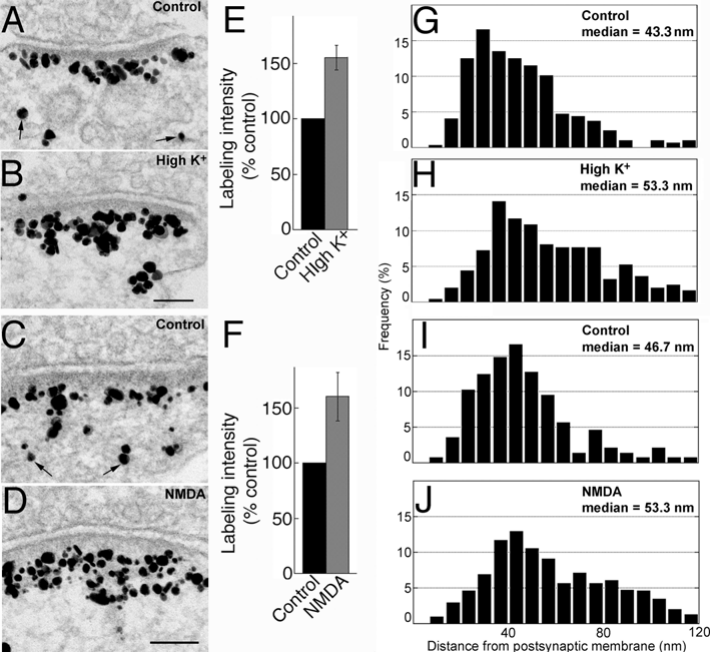
(A-D) Electron micrographs of asymmetric synapses from dissociated hippocampal cultures labeled for Shank3 using ab2. Scale bars = 0.1 μm (A and B share the same scale bar, C and D share the same scale bar). Labeling intensity increased upon 2 min treatment with either high K^+^ (B vs. A) or NMDA (D vs. C). (E, F) Bar graphs represent combined data (mean ± SEM) from S1 and S2 Tables. (G-J) Histograms from a typical experiment depicting frequency distribution of gold particles as a function of distance from the postsynaptic membrane. Median distance of label from postsynaptic membrane increased after high K^+^ (H vs. G) or NMDA treatment (J vs. I), indicating preferential recruitment of Shank3 molecules to the distal layer of the PSD.

To test whether Shank3 at the PSD also redistributes under excitatory conditions like Shank1 and Shank2 [7], hippocampal cultures were treated under different experimental conditions. Under basal conditions (Fig 4A), the bulk of the label for Shank3 was typically located in a broad band ~20–80 nm from the postsynaptic membrane (Fig 4G), mostly outside of the dense core of the PSD. Some particles were scattered further away in the cytoplasm (small arrows in Fig 4A). Upon depolarization with high K^+^ (90 mM, 2 min), labeling intensity of Shank3 at the PSD increased significantly (Fig 4B vs. 4A) to ~155% of control value (Fig 4E; S1 Table). Exposure to high K^+^ also resulted in a 10 nm increase in the median distance of label from the postsynaptic membrane over that of control (Fig 4H vs. 4G). These results are consistent with those for Shank1 and Shank2 [9], indicating that the additional Shank3 molecules recruited during high K^+^ treatment are preferentially located in the distal layer of the PSD.

Treatment with NMDA (50 μM, 2 min) also produced significant increases in labeling intensity for Shank3 at the PSD (Fig 4D vs. 4C; 4F) as well as in the median distance of label from the postsynaptic membrane (Fig 4J vs. 4I). These results are consistent with those reported for the pan-Shank antibody [8] where Shank was recruited to the distal layer of the PSD upon NMDA treatment.

### Zinc supplementation also increases Shank3 concentration at the PSD, but not preferentially at the distal layer of the PSD

**Fig 5:**
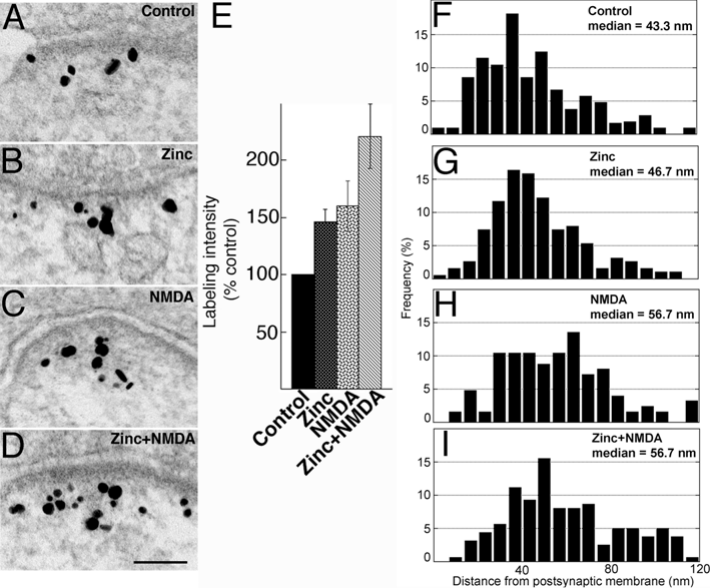
(A-D) Electron micrographs of asymmetric synapses labeled for Shank3 using ab1. Hippocampal cultures were pre-incubated for 1 hr in media with or without zinc and subsequently treated for another 2 min in the same media with or without NMDA, as indicated. After 1 hr zinc incubation (B) or 2 min NMDA treatment (C), labeling intensities were higher than that of control (A). Upon pre-incubation with zinc followed by NMDA treatment (D), labeling intensity was even higher than after either treatment alone. Scale bar = 0.1 μm. (E) Bar graphs represent combined data (mean ± SEM) from S2 Table. (F-I) Median distance of gold particles from the postsynaptic membrane increased upon NMDA treatment, either in the absence (H) or presence of zinc (I), but not in samples incubated with zinc only (G) when compared to control (F).

Incubation in medium containing 50 μM zinc chloride for 1 hr consistently increased labeling intensity for Shank3 at the PSD (Fig 5B vs. 5A) to ~145% of the control (Fig 5E; S2 Table). In contrast, labeling intensity for Shank1 was not affected by zinc treatment (101 ± 4% of controls in three experiments, S3 Table), while labeling intensity for Shank2 increased slightly to ~109% without reaching statistical significance (two experiments, S3 Table). These results are consistent with light microscopic observation that after zinc treatment, the intensity of synaptic puncta increased significantly for Shank3, but not for Shank1, and increased to a lesser degree for Shank2 [17]. These results also indicate that even if Shank3 antibodies used here cross react with Shank1 or Shank2, the contribution made by Shank1 or 2 is relatively minor, and that the bulk of the zinc-induced increase in labeling intensity of PSDs is attributable to Shank3.

Interestingly, zinc-induced increase of Shank3 label at the PSD was not preferential to the distal layer of the PSD. Laminar distribution and median distance of label after zinc incubation remained similar to that of control (Fig 5G vs. 5F; S4 Table), in contrast with those following treatment with NMDA (Fig 5H; S4 Table), or high K^+^ (Fig 4H; S1 Table), where the median distance of label from the postsynaptic membrane increased significantly.

When cells incubated with zinc for 1 hr were subsequently exposed to NMDA for 2 min (zinc+NMDA), labeling intensity of Shank3 at the PSD increased to ~220% of control (Fig 5E; S2 Table). The effects of the two reagents appeared to be additive as samples treated with both reagents typically had significantly higher labeling intensity than samples treated with either reagent alone (Fig 5E; S2 Table). Median distance of label from the postsynaptic membrane after zinc+NMDA treatment (Fig 5I) was significantly greater than those of control (Fig 5F) or zinc-treated samples (Fig 5G), while similar to that of NMDA-treated samples (Fig 5H; S4 Table).

### The zinc-induced increase of Shank3 label under basal conditions does not require NMDA receptor activation

In order to test whether the increased accumulation of Shank3 at the PSD in the presence of zinc depends on the activation of NMDA receptors, an NMDA receptor blocker (APV) was included in the incubation medium. Under basal conditions, APV did not block the zinc-induced Shank3 increase at PSD. Labeling intensity for Shank3 after zinc+APV incubation is similar to that of zinc incubation alone (S5 Table). These results indicate that under the basal condition, the increase of Shank3 at the PSD after 1 hr of zinc incubation can occur without NMDA receptor activation.

**Fig 6:**
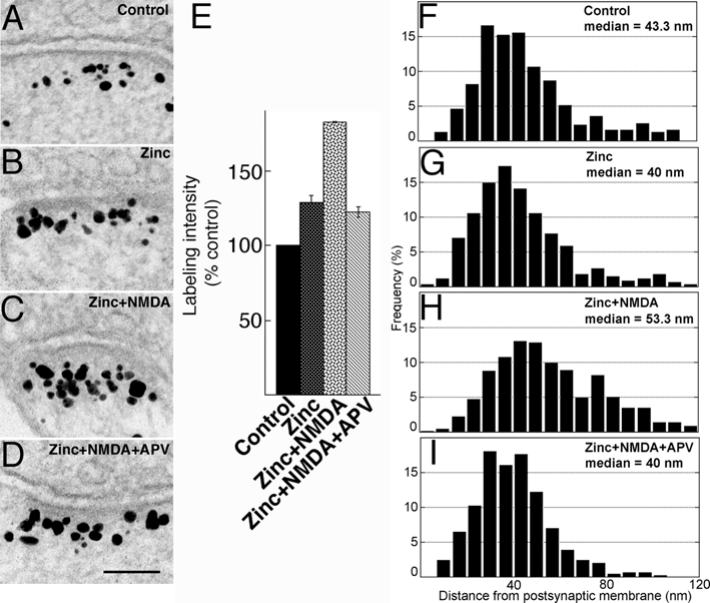
(A-D) Electron micrographs of asymmetric synapses labeled for Shank3 using ab2. Hippocampal cultures were pre-incubated for 1 hr in control medium or in media containing zinc or zinc+APV, followed by a 2 min treatment in the same media with or without NMDA, as indicated. Scale bar = 0.1 μm. (E) Bar graphs represent combined data (mean ± SEM) from S5 Table. APV appeared to block NMDA-induced, but not zinc-induced, increase in Shank3 labeling intensity at the PSD. (F-I) Laminar distribution and median distances of gold particles from the postsynaptic membrane were similar in control (F) and zinc-incubated samples (G). APV (I) blocked the increase in median distance in zinc+NMDA samples (H).

Upon acute NMDA treatment, APV effectively blocked the NMDA-induced recruitment of Shank3 to the PSD, but did not suppress the zinc-induced increase. The labeling intensity of Shank3 after zinc+NMDA+APV treatment (Fig 6D) was significantly lower than that after zinc+NMDA treatment (Fig 6C), but comparable to that of zinc incubation alone (Fig 6B), which, in turn, was significantly higher than that of control (Fig 6A; 6E; S5 Table). Inclusion of APV in the zinc+NMDA treatment also blocked NMDA-induced increase in median distance from the postsynaptic membrane (Fig 6I vs. 6H).

### Zinc does not alter the structure of the PSD or affect the distribution of CaMKII at the PSD

The detailed structure of the PSD was not affected by zinc supplementation either under basal or under excitatory conditions. After 1 hr incubation with zinc under basal conditions, no structural differences at the PSD were detected between zinc-incubated samples and controls (Fig 5B vs. 5A; Fig 6B vs. 6A). Upon acute NMDA and high K^+^ treatments, PSDs typically contained more associated dense materials and the PSDs changed curvature to arch into the presynaptic terminals (Figs 4B, 4D and 5C; Table 1), features that are consistent with activity-induced structural changes at the synapse [7, 20, 29]. Samples incubated with zinc for 1 hr and then treated with NMDA yielded PSDs structurally similar to those treated with NMDA alone (Fig 5D vs. 5C; Table 1). Thus, the effect of NMDA receptor activation on increasing the thickness and curvature of the PSD was present in samples treated with NMDA pre-incubated with zinc for 1 hr.

**Table 1:** Changes in index of curvature of PSD^a^.

	1. Control	2. Zinc	3. NMDA	4. Zinc+NMDA	5. Zn+N+APV
Exp 1	1.7 ± 1.1 (53)	2.5 ±0.9 (51) N. S. vs. 1	10.4 ± 1.4 (44) P<0.0001 vs. 1, 2	10.7 ± 1.6 (43) P<0.0001 vs. 1, 2; N. S. vs. 3	-
Exp 2	2.3 ± 0.9 (42)	1.1 ± 1.1 (45) N. S. vs. 1	7.6 ± 1.4 (34) P<0.05 vs. 1, P<0.001 vs. 2	8.2 ± 1.0 (47) P<0.001 vs. 1, P<0.0001 vs. 2, N. S. vs. 3	1.7 ± 1.0 (45) N. S. vs. 1, P<0.0001 vs. 4

^a^ Index of curvature of the PSD is calculated as the percent of the height of the arc of PSD / its length, and reported as mean ± SEM (n = number of synapses). One-way ANOVA within each experiment: N. S. (not significant)

**Fig 7:**
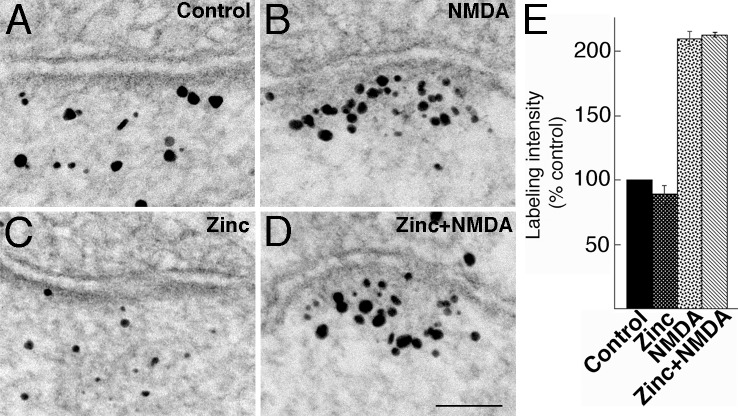
(A-D) Electron micrographs of asymmetric synapses labeled for CaMKII. Experimental conditions were as described in Fig 5. Label for CaMKII was dispersed in the postsynaptic cytoplasm under basal control conditions (A) or after zinc incubation (C), and the curvature of the PSD was typically flat. After NMDA (B) or zinc+NMDA (D) treatment, label for CaMKII was accumulated at the PSD, and the curvature of PSD was typically arched into the presynaptic terminal. Scale bar = 0.1 μm. (E) Bar graphs represent combined data from S6 Table. Zinc has no effect on the labeling intensity of CaMKII at the PSD either under basal conditions or upon treatment with NMDA.

Calcium calmodulin-dependent kinase II (CaMKII) is an abundant protein in glutamatergic neuronal spines, and is dispersed in the cytoplasm under basal conditions and aggregates at the PSD upon NMDA treatment [29]. To test a possible effect of zinc on the distribution of CaMKII, sister cultures were treated with zinc and/or NMDA, and labeled for CaMKII in parallel with Shank3. After zinc incubation, label for CaMKII was dispersed in the cytoplasm (Fig 7C), in a pattern similar to control samples (Fig 7A). After zinc+NMDA treatment, label for CaMKII became aggregated at the PSD and the curvature of the PSD arched into the presynaptic terminal (Fig 7D). These features of structural change as well as the pattern of redistribution for CaMKII resemble those in samples treated with NMDA alone (Fig 7B). These results again indicate that NMDA receptors were activated in samples pre-incubated with zinc for 1 hr. Furthermore, the intensity of labeling for CaMKII at the PSD did not change after zinc incubation under basal conditions or after exposure to NMDA (Fig 7E, S6 Table). Thus, in contrast to Shank3, the distribution of CaMKII at the PSD is not affected by incubation with zinc.

### NMDA-induced Shank3 accumulation at the PSD is not reversible in the presence of zinc

**Fig 8:**
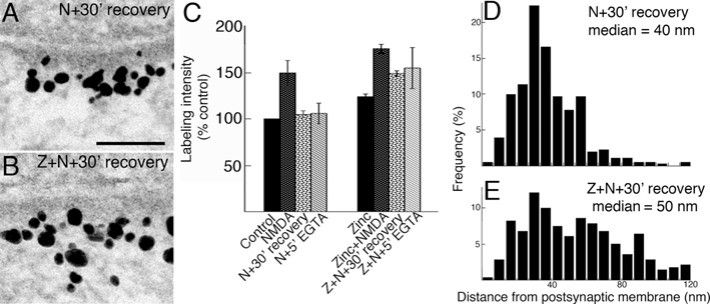
(A, B) Electron micrographs of asymmetric synapses labeled for Shank3 with ab2. Hippocampal cultures were pre-incubated for 1 hr with or without zinc, then exposed to NMDA for 2 min. Samples were either fixed immediately or after NMDA washout under two different protocols (30 min in control media or 5 min in EGTA-containing Ca^2+^-free media). Distribution patterns of label are different in samples that were incubated in the absence (A) or presence (B) of zinc, 30 min after NMDA washout. Scale bar = 0.1 μm. (C) Bar graphs represent combined data (mean ± SEM) from S7 Table. NMDA-induced increase of label for Shank3 is reversed in the absence of zinc, but largely retained in the presence of zinc. (D, E) Laminar distribution and median distances of gold particles from the postsynaptic membrane were also significantly different. NMDA-induced preferential increase of Shank3 in the distal layer of the PSD is reversible after 30 min recovery in control media (D), but maintained in the presence of zinc (E).

Shank3 distribution at the PSD returned to a pattern similar to that of control samples upon 30 min of recovery in control medium after NMDA treatment (Fig 8A). However, in parallel samples where zinc was included throughout the experiment, the NMDA-induced Shank3 accumulation at the PSD was largely retained including labeling located in the distal layer of the PSD (Fig 8B). Similar results were obtained with another protocol in which NMDA-treated samples were washed and kept in calcium-free medium containing EGTA for 5 min. Average values from three experiments show that labeling intensity for Shank3 at the PSD returned to control values after NMDA washout in the absence of zinc, but remained significantly higher in the presence of zinc (Fig 8C; S7 Table).

Laminar distributions of Shank3 30 min after NMDA washout were affected by zinc incubation as well. In one example, in the absence of zinc, median distance of label was 40 nm from the postsynaptic membrane, and the laminar distribution of label (Fig 8D) was similar to that of control (histogram not shown). In the presence of zinc, median distance of label 30 min after NMDA washout was significantly greater at 50 nm from the postsynaptic membrane, and the laminar distribution (Fig 8E) was similar to that in samples with acute NMDA treatment (histogram not shown). Thus, Shank3 molecules recruited to the distal layer of the PSD upon NMDA treatment are largely retained in the presence of zinc 30 min after NMDA washout.

## Discussion

It is difficult to be definitive about antibody specificity with Shanks since all three families of Shank proteins exhibit large numbers of splice variants [30]. However, we did get very similar results on cultured hippocampal neurons for Shank3 with two different antibodies (ab1 and ab2). We cannot completely eliminate the possibility that some of the labeling with Shank3 antibodies may be due to cross-reaction with Shank1 or Shank2, but a substantial portion of the label is contributed by Shank3.

Shank3, like its other two family members Shank1 and Shank2 [7], is located in a relatively broad layer extending beyond the electron dense core of the PSD, and shows an acute (within 2 min), activity-induced increase at the PSD. Interestingly, GKAP, the presumed binding partner of Shanks, is stably localized in a narrow layer close to the core of the PSD [9]. Thus, the three members of the Shank family reside in two pools at the PSD–one pool close to the core of the PSD where they can bind to GKAP, and a second pool in the distal layer of the PSD where Shanks are too far away to bind to GKAP [9]. It is in the distal layer of the PSD that the activity-induced accumulation of Shank preferentially occurs. Studies with a pan-Shank antibody indicate that the recruitment of Shanks to the PSD under acute excitatory conditions is calcium-dependent [7] and mediated by the Ca^2+^-regulated protein kinase CaMKII [8]. Furthermore, the distal pool of Shanks is preferentially depleted under low calcium conditions while the proximal GKAP-binding pool is not [9].

In addition to Shank-Shank associations [15], Homer tetramers that cross-link Shanks [31] may also play a role in tethering the proximal and distal pools of Shanks at the PSD. Homer co-localizes with Shank at the PSD under basal conditions but in contrast to Shank, Homer concentration and laminar distribution is not activity or calcium-dependent [22], or mediated by CaMKII [8]. Thus, Homer is likely not involved in the activity-dependent redistribution of Shanks. Other binding partners of Shank may play a role in activity-dependent response reflected in spine size and shape. Two likely candidates are Abp1 [27] and IRSp53 [32, 33], two actin regulators that may link the actin cytoskeleton to the PSD via Shank. The activity-dependent redistribution of Shanks may thus affect the organization of actin cytoskeleton in spines.

Supplementation of hippocampal cultures with zinc for 1 hr also increases the total amount of Shank3 at the PSD. However, unlike the activity-induced increase of Shank3 at PSD, the zinc-induced increase under basal conditions is not preferential to the distal layer of the PSD. Instead, Shank3 molecules are added throughout the PSD, assuming a laminar distribution resembling that of the control. Thus, zinc can indiscriminately promote accumulation of Shank3 molecules in both the proximal and the distal layers of the PSD.

The finding that zinc increases Shank3 concentration at the PSD in intact neurons could be explained by the *in vitro* demonstration that SAM domains of purified Shank3 associate into stacks that are stabilized by incubation with zinc [15]. Since all three members of the Shank family contain SAM domains, it is conceivable that zinc could strengthen association among Shanks of all types. However, a binding site for zinc is only present in the SAM domains of Shank3 and Shank2, but not in Shank1 [2]. Furthermore, after incubation with zinc, synaptic puncta increased in labeling intensity for Shank3 and Shank2, but not for Shank1 [17]. Thus, it is likely that the Shank-Shank interactions are stabilized by zinc for Shank3 and Shank2, but not for Shank1. Interestingly, super-resolution light microscopy reveals nanodomains within the PSD that exhibit a higher concentration of specific components [34, 35, 36] including Shank3 [34]. Stabilization of Shank-Shank association by zinc could contribute to the formation and maintenance of such nanoclusters.

Light microscopy of live cells expressing GFP-tagged Shanks at synaptic puncta shows fluctuating intensity over several hours [37], indicating that some Shanks come and go in dynamic flux at synapses under basal conditions. Because zinc stabilizes Shank associations, it is possible that more Shanks would be then retained at the PSD, resulting in a net increase of Shank with time. Here, we demonstrate that the zinc-induced increase of Shank3 under basal conditions is not suppressed by the NMDA antagonist APV, so mechanisms other than NMDA receptor activation must be involved in recruiting Shank3 to the PSD under basal conditions.

When cultures were pre-incubated with zinc and then treated with NMDA, even more Shank3 were recruited to the PSD. The concentration of zinc used in the present study (50 μM) has been shown to rapidly inhibit NMDA receptors by electrophysiological studies [38, 39]. However, in those studies, zinc was applied for only a few seconds immediately preceding NMDA application, an experimental protocol distinctly different from what is used in the present study where cultures were pre-incubated with zinc for 1 hr. Under our experimental condition, synapses clearly responded to application of NMDA with structural changes typical of stimulation, an indication that NMDA receptors are indeed activated under these conditions.

Without zinc supplementation, NMDA-induced accumulation of Shank3 at the PSD is transient. The labeling intensity and laminar distribution of Shank3 return to control levels within 30 min of removing NMDA. In contrast, when zinc is present under the same experimental conditions, the NMDA-induced accumulation of Shank3 at the PSD is largely retained. Most importantly, the laminar distribution of label for Shank3 remains similar to that in the NMDA-treated samples, with a sizable portion of label located in the distal layer of the PSD. These observations suggest that zinc also strengthens the association among Shank molecules throughout the PSD under excitatory conditions, causing them to be retained even after the episode of stimulation is over.

A scenario emerges involving the interplay of calcium-dependent and zinc-dependent processes in promoting an augmentation of the PSD scaffold through addition of Shanks. Synaptic activity induces a reversible translocation of Shanks to the PSD through a calcium-dependent process, while zinc, if present during activity-induced translocation of Shanks to the PSD, promotes stable associations among Shank molecules at the PSD.

While the signaling function of calcium is well documented, it is controversial whether zinc serves as a signaling element or as a trace element necessary for the maintenance of the PSD scaffold [10, 13]. Zinc is contained in synaptic vesicles in glutamatergic synapses and is released together with neurotransmitter upon synaptic activity, and the concentration of zinc in the synaptic cleft can reach the micromolar range [11, 40]. The amount of zinc that becomes available at the PSD during the critical window of calcium-mediated Shank accumulation could vary according to parameters such as the amount of zinc stored in vesicles, the strength and type of synaptic stimulation, availability of postsynaptic transport mechanisms for zinc, as well as how long zinc is retained in the PSD. There may be different situations where zinc and calcium would represent separate signaling mechanisms that ultimately converge to achieve and maintain activity-induced changes in the PSD scaffold.

## Supporting Information

S1 Table Changes in labeling intensity and median distance of label for Shank3 from the postsynaptic membrane after high K^+^. (DOCX)

S2 Table Labeling intensity for Shank3 at PSDs. (DOCX)

S3 Table Labeling intensity for Shank1 and Shank2 at PSDs. (DOCX)

S4 Table Median distance^a^ of label for Shank3 from the postsynaptic membrane. (DOCX)

S5 Table Effect of APV on labeling intensity of Shank3 under zinc and zinc + NMDA conditions. (DOCX)

S6 Table Effect of zinc on labeling intensity for CaMKII. (DOCX)

S7 Table Zinc effect on NMDA-induced increase in labeling intensity for Shank3 after washout of NMDA. (DOCX)
