# Supplementary material for: Zinc Stabilizes Shank3 at the Postsynaptic Density of Hippocampal Synapses
Source: PLoS One. 2016 May 4;11(5):e0153979. doi: 10.1371/journal.pone.0153979 (PMC4856407; doi:10.1371/journal.pone.0153979)
Supplement: S1 Table — (DOCX) [file pone.0153979.s001.docx]

**S1 Table. Changes in labeling intensity and median distance of label for Shank3 from the postsynaptic membrane after high K^+^**

| **Shank3 ab2** | **Labeling intensity^a^** | | **Median distance^b^** | |
| --- | --- | --- | --- | --- |
|  | **Control** | **High K^+^** | **Control** | **High K^+^** |
| **Exp 1** | 35.8 ± 2.0 (51) | 59.1 ± 3.5 (35) P<0.0001 | 46.7 (323) | 56.7 (259) P<0.0001 |
| **Exp 2** | 60.5 ± 2.7 (48) | 86.9 ± 3.7 (44) P<0.0001 | 43.3 (296) | 53.3 (248) P<0.0001 |
| **Combined mean ± SEM** | **100%** | **155 ± 11%**  **P<0.05** | **45 ± 1.7** | **55 ± 1.7**  **P<0.0001** |

^a^ Labeling intensity values are mean ± SEM expressed as number of labels /µm PSD, n = number of synapses (Student’s t test). Combined values in bottom row are means of two experiments normalized to control (paired t test).

^b^ Median distance from post synaptic membrane in nm, n=number of particles (Wilcoxon test). Combined values in bottom row are means of two experiments (paired t test).
