# Supplementary material for: Zinc Stabilizes Shank3 at the Postsynaptic Density of Hippocampal Synapses
Source: PLoS One. 2016 May 4;11(5):e0153979. doi: 10.1371/journal.pone.0153979 (PMC4856407; doi:10.1371/journal.pone.0153979)
Supplement: S2 Table — (DOCX) [file pone.0153979.s002.docx]

**S2 Table. Labeling intensity for Shank3 at PSDs**

|  | | **1. Control** | **2. Zinc** | **3. NMDA** | **4. Zinc+NMDA** |
| --- | --- | --- | --- | --- | --- |
| Ab1 | Exp 1 | 4.3 ± 0.6 (109) | 7.0 ± 0.8 (87) N. S. vs. 1 | 6.6 ± 0.9 (108) N. S. vs. 1 | 15.1 ± 1.7 (40) **** vs. 1, 2, 3 |
|  | Exp 2 | 10.2 ± 1.0 (91) | 16.1 ± 1.6 (71) ** vs. 1 | 9.7 ± 0.8 (135) N. S. vs. 1 | 14.5 ± 0.9 (188) * vs. 1,  N. S. vs. 2,  *** vs. 3 |
|  | Exp 3 | 4.9 ± 0.7 (95) | 5.4 ± 0.5 (153) N. S. vs. 1 | 8.3 ± 1.1 (75) * vs. 1 | 9.9 ± 1.1 (80) *** vs. 1, 2,  N. S. vs. 3 |
|  | Exp 4 | 4.5 ± 0.7 (83) | 9.5 ± 0.9 (88) ** vs. 1 | 13.8 ± 1.7 (45) **** vs. 1 | 15.2 ± 1.7 (70) **** vs. 1,  ** vs. 2,  N. S. vs. 3 |
|  | Exp 5 | 6.0 ± 0.8 (77) | 9.2 ± 0.8 (131) N. S. vs. 1 | 8.0 ± 1.5 (47) N. S. vs. 1 | 13.5 ± 1.5 (64) **** vs. 1,  * vs. 2, 3 |
| Ab2 | Exp 1 | 53.2 ± 3.0 (45) | 68.7 ± 3.0 (48) ** vs. 1 | 75.2 ± 2.6 (71) **** vs. 1 | 91.4 ± 3.7 (49) **** vs. 1, 2,  *** vs. 3 |
|  | Exp 2 | 59.1 ± 2.2 (80) | 71.1 ± 3.4 (57) * vs. 1 | 83.1 ± 3.4 (56) **** vs. 1 | 106.5 ± 3.5 (64) **** vs. 1, 2 , 3 |
|  | Exp 3 | 60.5 ± 2.7 (48) | 72.5 ± 3.2 (40) * vs. 1 | 81.9 ± 3.4 (45) **** vs. 1 | 98.9 ± 3.0 (45) **** vs. 1, 2,  ** vs. 3 |
| **Combined Mean ± SEM** | | **100**% | **146 ± 12% * vs. 1** | **160 ± 22% **** vs. 1** | **221 ± 28% **** vs. 1, 2,**  **** vs. 3** |

Labeling intensity values are mean ± SEM expressed as number of labels /µm PSD. (n = number of synapses)

Combined values in bottom row are means of all experiments normalized to control.

One-way ANOVA with Tukey’s post test: N. S. (not significant), *P<0.05, **P<0.01, ***P<0.001, ****P<0.0001.
