# Supplementary material for: Zinc Stabilizes Shank3 at the Postsynaptic Density of Hippocampal Synapses
Source: PLoS One. 2016 May 4;11(5):e0153979. doi: 10.1371/journal.pone.0153979 (PMC4856407; doi:10.1371/journal.pone.0153979)
Supplement: S3 Table — (DOCX) [file pone.0153979.s003.docx]

**S3 Table. Labeling intensity for Shank1 and Shank2 at PSDs**

|  | | | **1. Control** | **2. Zinc** | **3. NMDA** | **4. Zinc+NMDA** |
| --- | --- | --- | --- | --- | --- | --- |
| Shank1 | Exp 1 | | 14.9 ±1.3 (64) | 15.6 ± 1.4 (48) | - | - |
|  | Exp 2 | | 8.7 ±0.9 (30) | 9.1 ± 1.1 (35) | - | - |
|  | Exp 3 | | 8.6 ± 0.8 (32) | 8.0 ± 0.7 (34) | - | - |
|  | **Combined Mean±SEM** | | **100%** | **101 ± 4 %**  **N. S. vs. 1** |  |  |
| Shank2 | Exp 1 | | 22.0±1.4 (81) | 23.8±1.2 (131)  N. S. vs. 1 | 30.2±1.8 (78)  ** vs. 1 | 34.8±1.9 (82)  **** vs. 1, 2  N. S. vs. 3 |
|  | Exp 2 | | 18.1±1.2 (84) | 19.7±1.5 (62)  N. S. vs. 1 | 23.2±1.4 (81)  * vs. 1 | 29.7±1.6 (75)  **** vs. 1, 2  ** vs. 3 |
|  | | **Combined Mean±SEM** | **100**% | **108.5 ± 0.3% N. S. vs. 1** | **132.8 ± 4.6% ** vs. 1** | **161.2 ± 3.0% *** vs. 1, 2,**  **** vs. 3** |

Labeling intensity values are mean ± SEM expressed as number of labels /µm PSD. (n = number of synapses)

Combined values are means of all experiments normalized to control.

One-way ANOVA with Tukey’s post test: N. S. (not significant), *P<0.05, **P<0.01, ***P<0.001, ****P<0.0001.
