# Supplementary material for: Zinc Stabilizes Shank3 at the Postsynaptic Density of Hippocampal Synapses
Source: PLoS One. 2016 May 4;11(5):e0153979. doi: 10.1371/journal.pone.0153979 (PMC4856407; doi:10.1371/journal.pone.0153979)
Supplement: S4 Table — (DOCX) [file pone.0153979.s004.docx]

**S4 Table. Median distance^a^ of label for Shank3 from the postsynaptic membrane**

|  | | **1. Control** | **2. Zinc** | **3. NMDA** | **4. Zinc+NMDA** |
| --- | --- | --- | --- | --- | --- |
| Ab1 | Exp 1 | 46.7 (135) | 50.0 (128) N. S. vs. 1 | 60.0 (153) *** vs. 1, ** vs. 2 | 53.3 (125)  N. S. vs. 1, 2, * vs. 3 |
|  | Exp 2 | 46.7 (62) | 50.0 (117) N. S. vs. 1 | 53.3 (95)  N. S. vs. 1, 2 | 53.3 (273) * vs. 1,  N. S. vs. 2, 3 |
|  | Exp 3 | 43.3 (105) | 46.7 (190) N. S. vs. 1 | 56.7 (125) *** vs. 1, ** vs. 2 | 56.7 (161) **** vs. 1, 2,  N. S. vs. 3 |
| Ab2 | Exp 1 | 46.7 (283) | 40.0 (258) *** vs. 1 | 53.3 (549) **** vs. 1, 2 | 53.3 (390) **** vs. 1, 2,  N. S. vs. 3 |
|  | Exp 2 | 43.3 (393) | 40.0 (341) N. S. vs. 1 | 56.7 (478) **** vs. 1, 2 | 53.3 (677) **** vs. 1, 2,  N. S. vs. 3 |
| **Combined (Mean ± SEM)** | | **45.3 ± 0.8** | **45.3 ± 2.3 N. S. vs. 1** | **56.0 ± 1.3 *** vs. 1, 2** | **54.0 ± 0.1 ** vs. 1, 2,**  **N. S. vs. 3** |

^a^ Median distance in nm. (n = number of particles)

Combined values in bottom row are means of all experiments.

Median distances within each experiment were compared with Wilcoxon test, and values from all experiments (bottom row) were compared with one-way ANOVA with Tukey’s post test: N. S. (not significant), * P<0.05, **P<0.01, ***P<0.001, ****P<0.0001.
