# Supplementary material for: Zinc Stabilizes Shank3 at the Postsynaptic Density of Hippocampal Synapses
Source: PLoS One. 2016 May 4;11(5):e0153979. doi: 10.1371/journal.pone.0153979 (PMC4856407; doi:10.1371/journal.pone.0153979)
Supplement: S5 Table — (DOCX) [file pone.0153979.s005.docx]

**S5 Table. Effect of APV on labeling intensity of Shank3 under zinc and zinc + NMDA conditions**

|  | | **1. Control** | **Zinc** | | **Zinc+NMDA** | |
| --- | --- | --- | --- | --- | --- | --- |
|  |  |  | **2. No APV** | **3. with APV** | **4. No APV** | **5. with APV** |
| **Ab1** | **Exp 1** | 7.3 ± 0.6 (64) | 9.5 ± 0.5 (103) * vs. 1 | 9.6 ± 0.7 (67) * vs. 1 | - | - |
|  | **Exp 2** | 9.5 ± 0.6 (73) | 13.2 ± 0.8 (59) * vs. 1 | 11.9 ± 1.1 (44) | 17.4 ± 0.9 (82) **** vs. 1 | 12.0 ±0.7 (67) **** vs. 4 |
| **Ab2** | **Exp 1** | 53.2 ± 3.0 (45) | 68.7 ± 3.0 (48) *** vs. 1 | 64.2 ± 2.7 (52) * vs. 1 | - | - |
|  | **Exp 2** | 59.1 ± 2.2 (80) | 71.1 ±3.4 (57) * vs. 1 | - | 106.5 ±3.5 (64) **** vs. 1 | 73.9 ±2.7 (53) ** vs. 1,  **** vs. 4 |
| **Combined Mean ± SEM** | | **100%** | **129 ± 4.3 % **** vs. 1** | **126 ± 3.2 % *** vs. 1,**  **N. S. vs. 2** | **182.5±0.5 % **** vs. 1** | **122.5 ± 3.5 % ** vs. 1,**  **N. S. vs. 2, **** vs. 4** |

Labeling intensity values are mean ± SEM expressed as number of labels /µm PSD. (n = number of synapses)

Combined values in bottom row are means of all experiments normalized to control.

One-way ANOVA with Tukey’s post test: N. S. (not significant), * P<0.05, ** P<0.01, ***P<0.001, ****P<0.0001.
