# Supplementary material for: Zinc Stabilizes Shank3 at the Postsynaptic Density of Hippocampal Synapses
Source: PLoS One. 2016 May 4;11(5):e0153979. doi: 10.1371/journal.pone.0153979 (PMC4856407; doi:10.1371/journal.pone.0153979)
Supplement: S6 Table — (DOCX) [file pone.0153979.s006.docx]

**S6 Table. Effect of zinc on labeling intensity for CaMKII**

|  | **1. Control** | **2. Zinc** | **3. NMDA** | **4. Zinc+NMDA** |
| --- | --- | --- | --- | --- |
| Exp 1 | 32.3 ± 3.2 (38) | 31.1 ± 2.5 (42) N. S. vs. 1 | 69.4 ± 3.6 (45) | 68.3 ± 3.5 (48) |
|  |  |  |  | N. S. vs. 3 |
| Exp 2 | 32.8 ± 1.9 (49) | 26.8 ± 1.6 (57) N. S. vs. 1 | 66.9 ± 3.8 (64) | 70.6 ± 3.1 (76) N. S. vs. 3 |
| **Combined Mean ± SEM** | **100%** | **89 ± 7%** | **210 ± 6%** | **213 ± 2%** |

Labeling intensity values are mean ± SEM expressed as number of labels /µm PSD. (n = number of synapses). One-way ANOVA with Tukey’s post test: N. S. (not significant).

Combined values in bottom row are means of all experiments normalized to control.
