# Supplementary material for: Zinc Stabilizes Shank3 at the Postsynaptic Density of Hippocampal Synapses
Source: PLoS One. 2016 May 4;11(5):e0153979. doi: 10.1371/journal.pone.0153979 (PMC4856407; doi:10.1371/journal.pone.0153979)
Supplement: S7 Table — (DOCX) [file pone.0153979.s007.docx]

**S7 Table. Zinc effect on NMDA-induced increase in labeling intensity for Shank3 after washout of NMDA**

|  | |  | **Control** | **NMDA** | **NMDA**  **+30’recovery** | **NMDA**  **+5’ EGTA** |
| --- | --- | --- | --- | --- | --- | --- |
| ab1 | Exp 1 | Without zinc | 6.7 ± 1.0 (85) | 10.1 ± 1.0 (99) | 7.7 ± 1.0 (70) | 7.8 ± 1.5 (49) |
|  |  | with zinc | 8.8 ± 1.1 (70) | 12.4 ± 1.3 (74) | 10.4 ± 1.0 (79) | 11.8 ± 1.4 (75) |
| ab2 | Exp 1 | Without zinc | 58.2 ± 2.1 (60) | 100.0 ± 4.1 (52) | 60.8 ± 2.9 (70) | – |
|  |  | with zinc | 71.2 ± 3.1 (49) | 100.1 ± 4.7 (46) | 85.4 ± 2.7 (73) | – |
|  | Exp 2 | Without zinc | 49.4 ± 2.5 (78) | 62.5 ± 2.3 (91) | 52.0 ± 2.5 (64) | 46.9 ± 2.7 (78) |
|  |  | with zinc | 59.5 ± 2.5 (75) | 83.8 ± 3.5 (61) | 71.5 ± 2.4 (85) | 65.5 ± 3.0 (68) |
| **Combined Mean ± SEM** | | Without zinc | **1. 100%** | **2. 150 ± 13%** | **3. 108 ± 4% P<0.05 vs. 2** | **4. 106 ± 11% P<0.05 vs. 2** |
|  |  | with zinc | **5. 124 ± 3%** | **6. 176 ± 5%** | **7. 149 ± 3% P<0.05 vs. 3** | **8. 155 ± 22% P<0.05 vs. 4** |

Labeling intensity values are mean ± SEM expressed as number of labels /µm PSD. (n = number of synapses)

Combined values in bottom two rows are means of all experiments normalized to control without zinc. One-way ANOVA with Turkey’s post test.
